# Pancreatic Arteriovenous Malformation Presenting With Gastrointestinal Bleeding and Treated by Transarterial Embolization

**DOI:** 10.7759/cureus.100386

**Published:** 2025-12-30

**Authors:** Helen Bolanaki, Ioannis Tzimagiorgis, Hippocrates Moschouris, Savvas P Deftereos, Anastasios J Karayiannakis

**Affiliations:** 1 Second Department of Surgery, University Hospital of Alexandroupolis, Alexandroupolis, GRC; 2 Department of Radiology, Democritus University of Thrace, University Hospital of Alexandroupolis, Alexandroupolis, GRC; 3 Second Department of Surgery, Democritus University of Thrace, University Hospital of Alexandroupolis, Alexandroupolis, GRC

**Keywords:** arterial embolization, gastrointestinal bleeding, gastrointestinal endoscopy, pancreatic arteriovenous malformation, selective angiography

## Abstract

Arteriovenous malformation is a rare disease characterized by abnormal vascular connections between arteries and veins. The condition may remain asymptomatic until complicated by hemorrhage. We present a case of arteriovenous malformation in the pancreatic head complicated by upper gastrointestinal bleeding, which was successfully treated by selective transarterial embolization. A 62-year-old man presented with mild epigastric pain and several episodes of melena. Colonoscopy and upper gastrointestinal endoscopy were not diagnostic. Contrast-enhanced computed tomography revealed abnormal enhancement with multiple vascular branches in the pancreatic head, with early enhancement of the portal venous system in the arterial phase, suggesting the presence of an arteriovenous malformation. Selective angiography revealed an enlarged gastroduodenal artery feeding the malformation and subsequently draining into the portal venous circulation. Embolization of the gastroduodenal artery and of other small branches from the superior mesenteric artery resulted in complete obliteration of the feeding arteries and drainage veins. There was no bleeding recurrence, and the epigastric pain resolved. This case report demonstrates that selective transarterial embolization is a feasible and effective procedure for the treatment of a bleeding pancreatic head arteriovenous malformation.

## Introduction

Arteriovenous malformation (AVM) represents an anomalous network of blood vessels that causes an aberrant communication between arteries and veins, resulting in irregular perfusion of tissues. The condition is usually congenital and most often occurs in the brain and the spinal cord, but it may develop anywhere in the body. AVMs have been described in the gastrointestinal tract, usually in the large and small intestine, but also in solid organs such as the pancreas [1,2].

AVMs are usually asymptomatic or associated with vague symptoms depending on their location. Hemorrhage is the most severe complication of AVMs, with symptoms related to the severity of bleeding and the organ involved [3].

Here, we present an unusual case of upper gastrointestinal hemorrhage caused by an AVM of the pancreatic head, which was treated by selective transarterial embolization.

## Case presentation

A 62-year-old man presented with mild, non-colicky, non-radiating epigastric pain and several episodes of melena over the past three days. On admission, he was apyrexial, his blood pressure was 110/65 mmHg, and his heart rate was 86 beats per minute. His medical history included a laparoscopic cholecystectomy two weeks before and arterial hypertension under treatment with felodipine.

**Figure 1:**
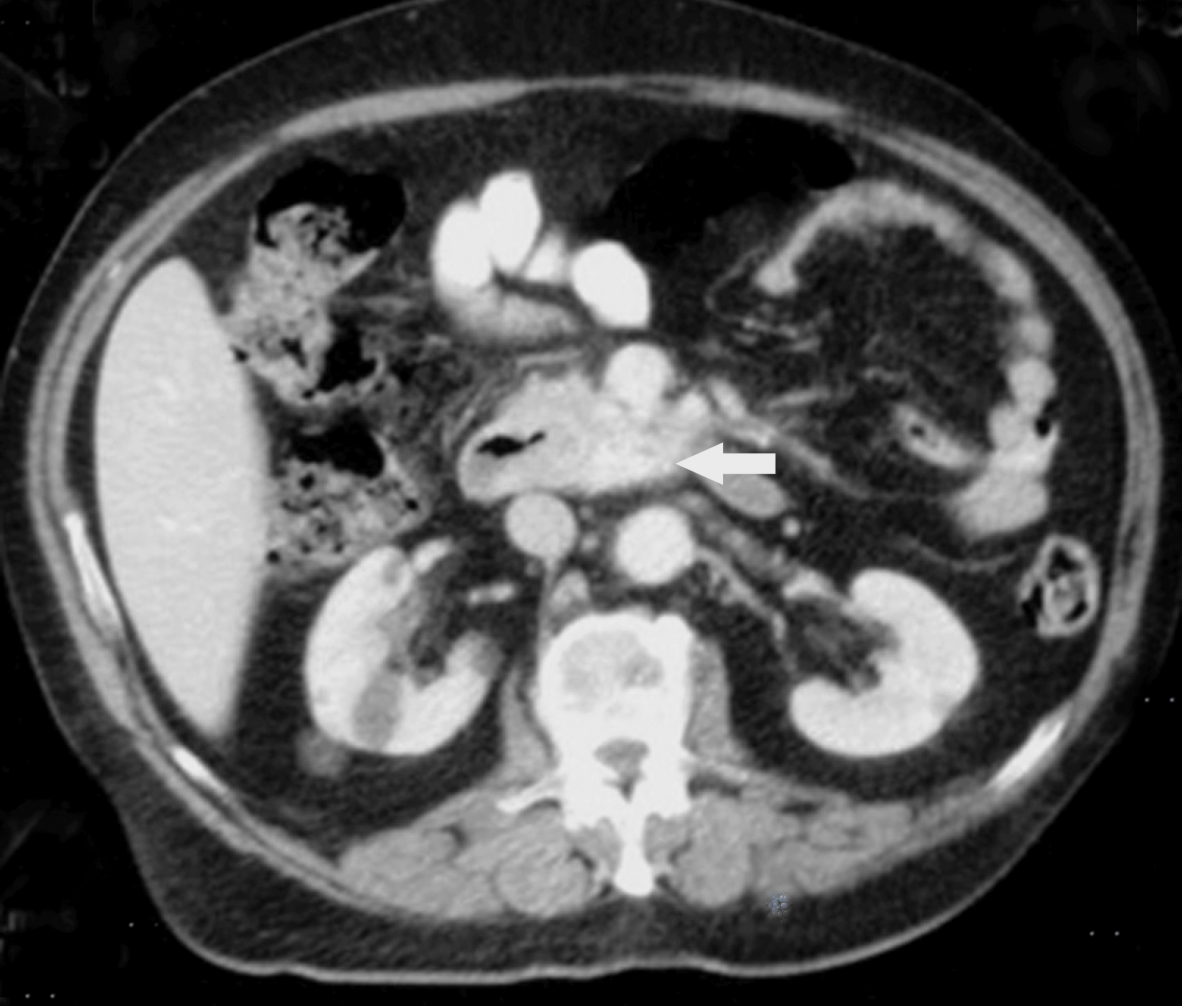
Contrast-enhanced computed tomography of the abdomen. Showing increased vascularity in the pancreatic head and the uncinate process (arrow).

**Figure 2:**
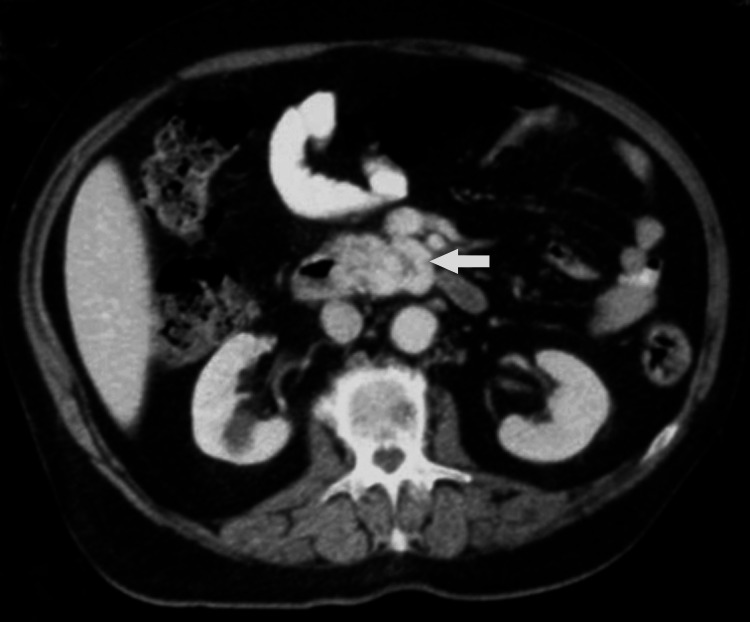
Contrast-enhanced computed tomography of the abdomen. Showing a prominent draining vein coursing towards the superior mesenteric vein (arrow).

Blood analysis showed anemia with a hemoglobin level of 5.6 g/dL (reference range: 11.0-15.0 g/dL) and a hematocrit of 19%. After resuscitation with colloids and three units of packed red blood cell transfusion, the patient underwent a colonoscopy, which was unremarkable. Upper gastrointestinal endoscopy revealed black flat spots in the medial border of the second and third parts of the duodenum, but not active bleeding, visible vessels, or ulcer. These findings probably reflected duodenal mucosal ischemia or submucosal hemorrhage due to steal of flow into the adjacent pancreatic head AVM. Abdominal contrast-enhanced computed tomography showed abnormal enhancement with multiple vascular branches within the pancreatic head and the uncinate process bordering the third part of the duodenum (Figure 1). There was early, dense opacification of the draining veins during the arterial phase, consistent with arteriovenous shunting. A prominent draining vein was seen coursing toward the superior mesenteric vein (Figure 2), with premature enhancement of the portal venous system. The diagnosis of an AVM was made.

**Figure 3:**
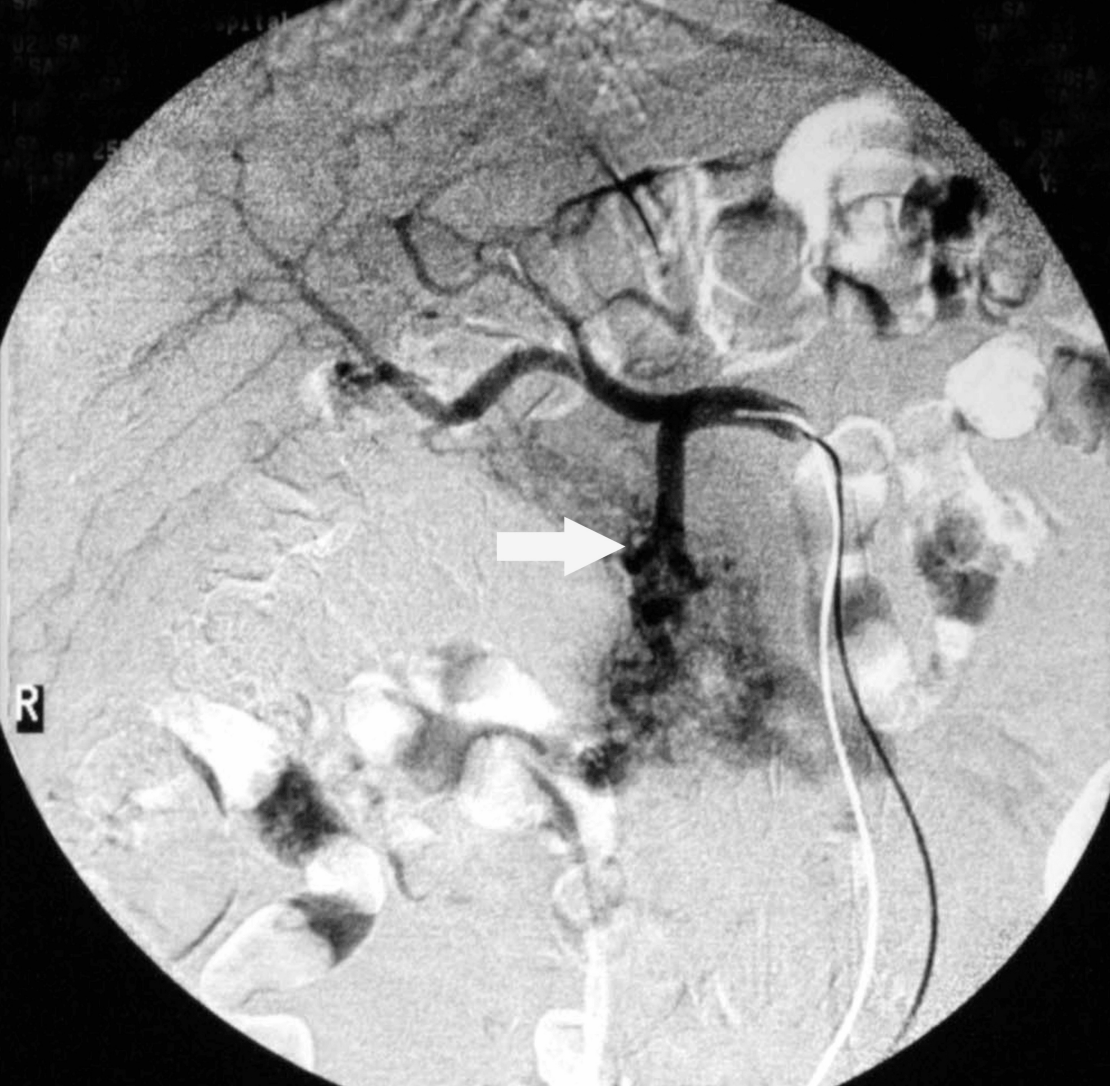
Selective arteriography of the common hepatic artery. An enlarged gastroduodenal artery feeding the malformation is seen (arrow).

**Figure 4:**
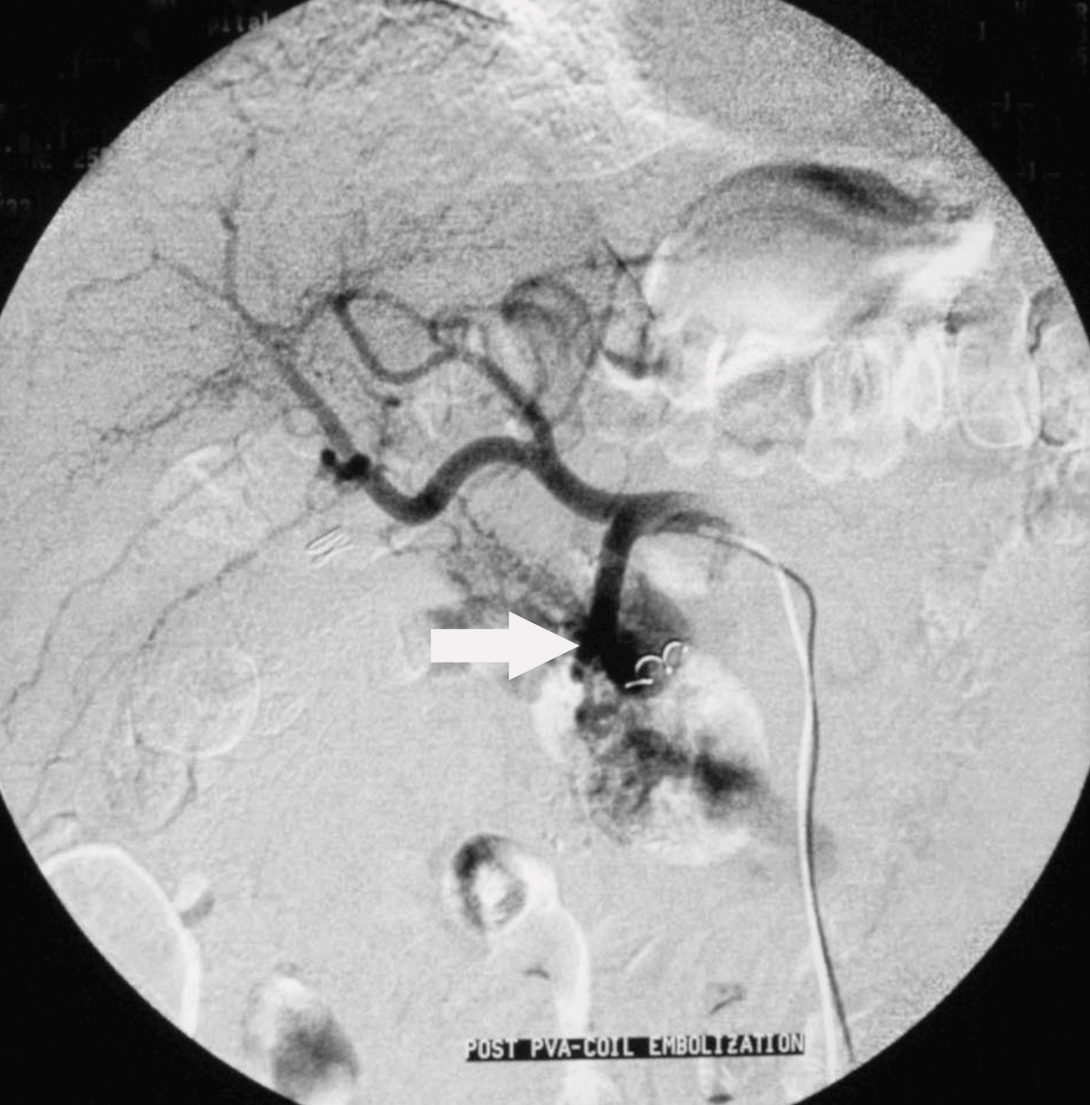
Selective embolization of the gastroduodenal artery. Post-embolization arteriography of the gastroduodenal artery shows no further feeding of the malformation (arrow).

Selective catheterization and arteriography of the common hepatic artery revealed an enlarged gastroduodenal artery feeding the malformation with early visualization of the splenic and portal veins (Figure 3). Embolization of the gastroduodenal artery with 1,000 μm microspheres was successful, and the visualization of the portal vein was terminated. Additional small arterial branches from the superior mesenteric artery draining into the splenic vein were occluded with coils. Post-embolization arteriography of the gastroduodenal artery and of the superior mesenteric artery revealed no draining into the portal and splenic veins (Figure 4). No major complication was encountered. There was no bleeding recurrence, the epigastric pain resolved, and the patient was discharged five days later. He remains well and symptom-free two years after embolization.

## Discussion

AVM of the pancreas represents an abnormal vascular network that causes an aberrant communication between arteries and veins. Pancreatic AVMs are rare (0.9%); the splenic artery is most commonly involved (42%), followed by the gastroduodenal artery (22%) and small pancreatic arterial branches (25%) [1]. They are most commonly located in the pancreatic head (63.5%), as in this case, followed by the pancreatic tail (24.8%) and body (22.8%) [4] or may involve the entire pancreas (8.9%) [5] diffusely. This condition is usually congenital (90%) but may develop secondary to pancreatitis, trauma, or neoplasm and affects males more frequently (87%) at a median age of 50 years [4].

AVMs are asymptomatic in 5.1% of cases [4] and are incidentally detected by imaging studies performed for other reasons or may be associated with a variety of symptoms such as pain, duodenal ulcer, jaundice, pancreatitis, and portal hypertension [1,2,6]. Abdominal pain is the most common symptom (50.5%), followed by gastrointestinal bleeding (42.7%), pancreatitis (22.2%), duodenal or gastric ulcer (11.1%), and portal hypertension (6.8%) [4]. Hemorrhage is the most severe complication of pancreatic AVMs with a mortality rate of 30-50% [5]. Gastrointestinal bleeding may occur through the pancreatic duct (hemosuccus pancreaticus) [1], the bile duct (hemobilia), the duodenal mucosa (ulcer), or from esophagogastric varices in case of portal hypertension development [7-10]. It has been reported that bleeding occurs more often (62.7%) when the AVM is located in the pancreatic head compared to other locations (26.7%) [2]. The symptoms relate to the severity of bleeding. In cases of pancreatic head AVM, there is usually intermittent or massive upper gastrointestinal hemorrhage from the duodenal mucosa. Endoscopy is less diagnostic because active bleeding or the source of bleeding is not always apparent [7,11]. In our case, the observed “black flat spots” in the duodenum during upper endoscopy may represent duodenal mucosal ischemia or submucosal hemorrhage secondary to the vascular "steal" phenomenon caused by the adjacent pancreatic head AVM, and should be considered as a worrying feature by the endoscopists encountering similar subtle findings. However, a bleeding ulcer in the second part of the duodenum has been reported [8,9], probably as a result of mucosal ischemia and ulceration due to this phenomenon [4].

Contrast-enhanced CT is a valuable diagnostic tool. Typically, it reveals an abnormal vascular network in the pancreatic head supplied by arterial branches from the gastroduodenal artery and/or the superior mesenteric artery and early contrast filling during the arterial phase of the portal venous system, splenic and/or superior mesenteric vein [5,11]. Similar findings can be demonstrated by magnetic resonance imaging showing multiple “honeycomb”-like flow voids in all sequences, which represent the abnormal vascular network [3,6,11]. Digital subtraction angiography is the gold standard for the diagnosis of pancreatic AVM and has been used in most reported cases [2,3,6-8,10,11]. Typically, angiography reveals a complex intrapancreatic vascular network along with the dilated feeding arteries and the early draining veins. It is also helpful for excluding other hypervascular pancreatic lesions such as islet cell neoplasms, cystadenocarcinoma, sarcoma, or metastases, and in predicting the treatment options.

Surgical resection of the pancreatic portion bearing the malformation has been suggested as an effective treatment for pancreatic AVMs and has been used in 44.3% of reported cases [4]. Treatment of a bleeding pancreatic head AVM by pylorus-preserving pancreaticoduodenectomy or pancreaticoduodenectomy has been described in more than two-thirds of reported cases with or without previous selective arterial embolization to reduce intraoperative blood loss [3,4,8,12-14]. Patients with body or tail AVMs were treated by distal pancreatectomy or by total pancreatectomy when the entire pancreas was involved [1,6].

In our patient, transarterial embolization was chosen as the first-line treatment of this benign condition in order to avoid a major operation such as pancreaticoduodenectomy, which is associated with high postoperative morbidity and mortality. The favorable anatomy of the AVM with clear identification of the feeding artery also contributed to this decision. It is conceivable that other factors, such as the patient’s age, the presence of comorbidities, the available facilities, and the experience of the interventional radiologist, might influence the decision for transarterial embolization. Nowadays, selective transarterial catheterization and embolization are the preferred method of treatment, especially in high-risk patients, with an overall success rate of 57.7% [4]. Embolization is most effective (80%) in small-sized AVMs with a few feeding arteries and drainage veins and no portal hypertension. Large lesions are best treated by surgical resection with or without previous embolization [15-17]. The treatment of patients with asymptomatic AVMs is not clear. Successful conservative treatment has been reported for asymptomatic patients, patients not fit for surgery, or those presenting with pain only [5,7]. However, they should be followed clinically by color Doppler ultrasound and/or CT-angiography [6,16].

## Conclusions

AVM of the pancreatic head should be considered in cases of upper gastrointestinal bleeding, particularly when there are no mucosal endoscopic findings. Contrast-enhanced CT is a useful diagnostic tool. However, selective transarterial catheterization is more accurate diagnostically and can be combined with embolization for the successful treatment of this condition, especially in high-risk patients.

## References

[1] Song KB, Kim SC, Park JB (2012). Surgical outcomes of pancreatic arteriovenous malformation in a single center and review of literature. Pancreas.

[2] Chou SC, Shyr YM, Wang SE (2013). Pancreatic arteriovenous malformation. J Gastrointest Surg.

[3] Wu W, An FD, Piao CL (2021). Management of pancreatic arteriovenous malformation: case report and literature review. Medicine (Baltimore).

[4] Onozawa S, Miyauchi R, Takahashi M, Kuroki K (2023). An update of treatment of pancreatic arteriovenous malformations. Interv Radiol (Higashimatsuyama).

[5] Hansen W, Maximin S, Shriki JE, Bhargava P (2015). Multimodality imaging of pancreatic arteriovenous malformation. Curr Probl Diagn Radiol.

[6] Seike T, Komura T, Shimizu Y (2019). A case of chronic pancreatitis exacerbation associated with pancreatic arteriovenous malformation: a case report and literature review. Clin J Gastroenterol.

[7] Jana T, Machicado JD, Guha S (2014). Gastrointestinal bleeding caused by pancreatic arteriovenous malformation. Clin Gastroenterol Hepatol.

[8] Arora A, Tyagi P, Kirnake V (2013). Unusual cause of massive upper gastrointestinal bleeding: a pancreatic arteriovenous malformation. JOP.

[9] Takayama H, Shimodate Y, Nomura S (2020). Bleeding from pancreatic arteriovenous malformation with duodenal ulcer penetration. Case report and literature review. Clin Case Rep.

[10] Kato S, Kobayashi N, Kubota K (2010). A duodenal mucosal lesion coming from pancreatic arteriovenous malformation. Gastrointest Endosc.

[11] Nikolaidou O, Xinou E, Papakotoulas P, Philippides A, Panagiotopoulou-Boukla D (2018). Pancreatic arteriovenous malformation mimicking pancreatic neoplasm: a systematic multimodality diagnostic approach and treatment. Radiol Case Rep.

[12] Kitazono M, Fujita M, Katsue K (2021). A case of emergency pancreatoduodenectomy for bleeding from the duodenal mucosa due to arteriovenous malformation of the pancreatic head. Clin Case Rep.

[13] Korai T, Kimura Y, Imamura M (2020). Arteriovenous malformation in the pancreatic head initially mimicking a hypervascular mass treated with duodenum-preserving pancreatic head resection: a case report. Surg Case Rep.

[14] Hakoda H, Kawaguchi Y, Miyata Y, Togashi J, Nagai M, Suzuki Y, Nomura Y (2022). Surgical resection of arteriovenous malformation of the pancreatic head with acute pancreatitis: a case report. J Surg Case Rep.

[15] Shin SH, Cho CK, Yu SY (2023). Pancreatic arteriovenous malformation treated with transcatheter arterial embolization: two case reports and review of literature. World J Clin Cases.

[16] Marcelin C, Park AW, Gilbert P (2022). Management of pancreatico-duodenal arterio-venous malformation. CVIR Endovasc.

[17] Yoon SY, Jeon GS, Lee SJ, Kim DJ, Kwon CI, Park MH (2020). Embolization of pancreatic arteriovenous malformation: a case report. World J Clin Cases.

